# Practical Observations from an Epidemiological Investigation of a Measles Outbreak in a District of India

**DOI:** 10.4103/0970-0218.51234

**Published:** 2009-04

**Authors:** Ashok Mishra, Subodh Mishra, Chandrakant Lahariya, Pankaj Jain, Rahul S Bhadoriya, Dhiraj Shrivastav, Neera Marathe

**Affiliations:** Department of Community Medicine, Gajara Raja Medical College, Gwalior, MP - 474 009, India

**Keywords:** Attack rate, case fatality rate, measles epidemic, post measles complication, vaccine failure

## Abstract

**Background:**

Measles is a major cause of childhood morbidity and mortality, accounting for nearly half of the morbidity associated with global vaccine preventable diseases. Regular outbreaks of Measles are reported in India, of which only a few are investigated. This study was conducted in the Shivpuri District of Madhya Pradesh (India) to investigate and asses various epidemiological factors associated with measles outbreak.

**Materials and Methods:**

A cross-sectional study was carried out in 30 randomly selected sub-centers in 8 blocks of the Shivpuri District of Madhya Pradesh, covering 212 villages, selected by cluster sampling. The villages, which had reported measles cases, were extensively investigated by the field teams through extensive house-to-house surveys during 12-19 May 2004.

**Results:**

A total of 1204 cases with 14 deaths were reported with an attack rate of 6.2% and a case fatality rate of 1.2%. In this study, 17.7% of the cases reported post-measles complications with diarrhea as the most common post measles complication. The routine measles vaccine and Vitamin A supplementation in the area was also less than 30%.

**Conclusions:**

The majority of the cases had occurred in the unvaccinated children and in under 5 year old population. There are repeated outbreaks and a long delay in reporting of the cases. The occurrence of cases, in a reasonable proportion of the vaccinated population, points toward the fact that there is a possibility of a vaccine failure in older children. This study calls for an improved surveillance system, an improvement in the cold chain, and enhancements for measles vaccination if India is to achieve the goal of measles elimination.

## Introduction

Measles is one of the leading causes of childhood morbidity and mortality in the world despite the availability of a safe, effective, and relatively inexpensive vaccine.(1) The children in developing countries are the main victims.(2) The World Health Organization (WHO) estimates that almost one million measles-related deaths occur each year, the majority (85%) in Africa and Asia.(3) In May 2003, the World Health Assembly adopted the goal for achieving a reduction of measles deaths by half by the year 2005 compared with the year 1999.(1) In India, though overall immunization levels are high, many districts have coverage far below the national average. Vaccine coverage varies within the different states, which leads to a pool of vulnerable target groups that are susceptible to the disease.(4) Moreover, studies from the rural, semi-urban, slum, and community revealed poor vaccine coverage along with low vaccine efficacy(5) leading to a susceptible population in which outbreaks occur.(6–8)

An outbreak of measles was reported in Shivpuri district of Madhya Pradesh in April 2004 with little mortality. To better understand the scale of the outbreak, Madhya Pradesh Department of Health and Family Welfare requested the Department of Preventive and Social Medicine (PSM)/Community Medicine, Gajra Raja Medical College, Gwalior (India) to conduct an outbreak investigation in the district of Shivpuri to give recommendations for further action. Therefore, this study was undertaken to investigate the extent of the problem, possible factors responsible for its occurrence and to institute preventive and control measures.

## Materials and Methods

**Study area:** Shivpuri is one of the border districts in Madhya Pradesh, adjoining both Uttar Pradesh and Rajasthan. The district headquarters of Shivpuri is located at National Highway-3 (Agra-Bombay National Highway), 113 kms from the district of Gwalior and has an estimated population of 1,441,950 with 1,202,277 of the population (83.3%) being rural.(9) The district is divided into eight blocks namely, Badarwas, Karera, Khaniadhana, Kolaras, Narwar, Pichore/Manpura, Pohari, and Satanwada.

**Study design:** A cross-sectional survey was carried out from May 12-19, 2004 by ten teams from the Department of Community Medicine with technical support from UNICEF. The teams comprised of faculty members, post-graduate students, and interns from the Department of Community Medicine and the paramedical staff from Shivpuri District. Although no intervention was planned in this study, the approval from the institutional ethics committee was taken and it was decided that any identified case will be provided necessary treatment at the earliest possible time.

**Sampling:** The rural area of Shivpuri had 199 sub-centers in 2004. Considering the time and resources available, a convenient sample of 30 sub-centers was taken for this study. The methodology used in 30 cluster techniques for immunization coverage was appropriately adapted for the selection of sub-centers in the current study. (A complete list of sub-centers was made along with the population of individual sub-centers and the cumulative population. A sampling interval was calculated by dividing the total population by 30. A number less than the sampling interval was taken for the selection of the first study sub-center and the subsequent sub-centers were selected by adding sampling intervals until 30 sub-centers were selected. Sub-centers selected in this way were included in this study. A total of 212 villages were covered under these 30 sub-centers. The contact was made with the village leaders/members to seek their cooperation for the investigation. A total of 206 villages were surveyed by an investigating team in 30 sub-centers who reported having measles cases. (The remaining 6 villages could not be covered either due to inaccessibility or non cooperation from village members).

The standard case definition was used for diagnosis of measles. A combination of major and minor criteria was used to clinically identify the measles cases.

### Major criteria

- A child with a high fever (101°C) for 3 days or more
- Characteristic rashes that started behind the ears

### Minor criteria

- Presence of cough, coryza, or conjunctivitis

A study subject was considered to have measles if he presented with one of the major criteria and any of the three minor criteria.(3)

Only the clinical definition of measles (mentioned above) was considered as the index case was already evaluated by experts. The household was a sampling unit and the villages were extensively covered by house-to-house visits made by the research teams. All the households in a single village were covered. With regard to the fever or rash cases in the household in the last 3 months, if any such case was found, extensive information was collected for that study subject. A total population of 193,000 was covered under the investigation to determine the measles cases.

Enquiries about measles cases were made from the mothers or responsible persons. Relevant information was collected on a WHO standard questionnaire modified to suit local needs. Information was collected regarding age, gender, history of measles in the last 3 months, immunization status against measles before the illness, and post-measles complications. A child who had received a measles vaccine before the current attack of measles was considered to be an immunized child in this study. Immunization status was assessed by checking the cards where available or by a convincing history of immunization given by the mothers. “Unknown” was used to designate patients with no knowledge of their immunization status. If any measles cases were identified by the above definition, an inquiry was made about the complications on the basis of history. Any episode of diarrhea, pneumonia, ear infection (dummy for Ottitis media), or any identified complication, which led to the hospitalization of a subject was considered to be a complicated case for this study. Both active and recovered cases since the beginning of year 2004 were included in the study. The data so collected was analyzed using EPI-Info (version 6) software.

## Results

The present study was conducted in 206 villages of 30 sub-centers covering a population of 193,931. A total of 1204 measles cases and 14 deaths due to measles were reported until May 2004. Data regarding measles morbidity and mortality for the last 3 years was reviewed to ascertain the possibility of an epidemic. The information on the reported measles cases in previous years and until April 2004 was collected. The analysis revealed this to be an epidemic as there was a clear excess of cases in comparison with the previous 3 years. (Data not included in this article.) The break-up of the cases by the blocks was also collected and it was noted that the highest proportion of cases were reported in the Pichore and Karera block while the attack rate was the highest in Pichore block.

**Figure 1:**
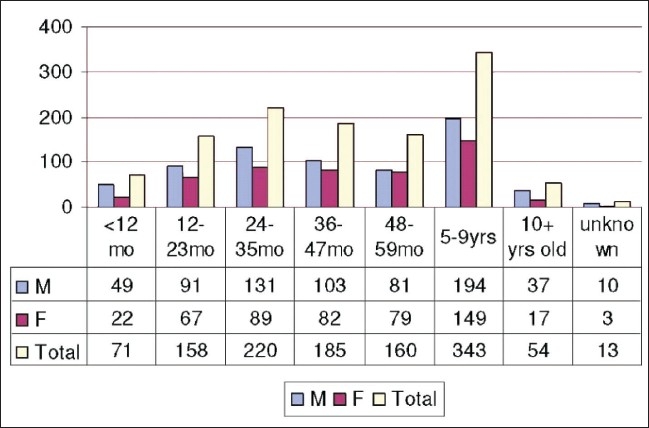
Age and gender distribution of measles cases

Out of 1204 cases, 696 (57.8%) were males and 508 (42.2%) were females. A higher proportion of measles was observed in male children compared with females. However, this difference was not found to be statistically significant. A majority of the cases (65.9%) were recorded in children under the age of 5. The age of the cases ranged from 4 weeks to 25 years. The age and gender distribution of cases is given in Figure 1.

Two peaks of age for measles were observed, the first was among 2-3 year olds and the second was among 5-9 year olds. The overall attack rate was found to be 6.2% in the study population. The attack rate among children under the age of 5 years was estimated to be 24.2%.This difference was found to be highly significant when compared with the rest of the affected population (*P*<0.001). The overall attack rate and the attack rate for children under the age of 5 are shown in Table 1.

**Table 1:** Measles attack rate in the study population

Population	Cases	Attack	Rate (%)
Total sample	(193,931)	1204	06.2
Among children aged less than	(32,774)	794	24.2
5 years			

**Figure 2:**
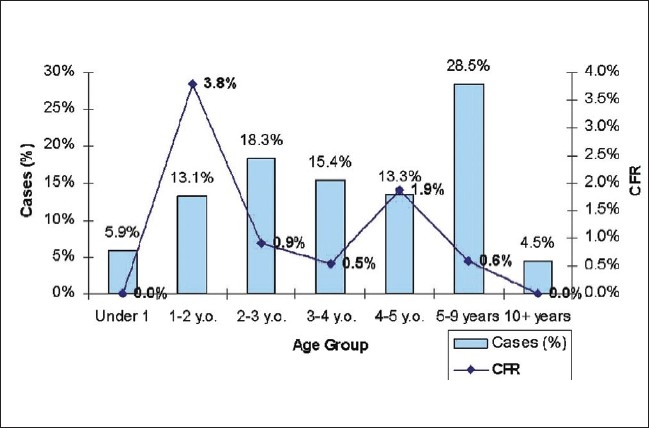
Age wise case fatality rate in the study population

Out of the 1204 cases, 14 were fatal (case fatality rate [CFR] of 1.2%). However, the variation in CFR by age presented in Figure 2 was substantial. The CFR was highest among children who were 1-2 years old. The CFR in males was 0.9% (696 cases, 6 deaths), while in females, it was 1.6% (508 cases, 8 deaths). Table 2 shows the age specific measles fatality rate. The females experienced a higher CFR in comparison with males. However, this difference was not statistically significant.

**Table 2:** Age specific measles fatality rates

Age group	Cases	Deaths	CFR	Complications occurred no. (%)
<12 months	71	0	0	4 (05.6)
12-23 months	158	6	3.8	38 (24.0)
24-35 months	220	2	0.9	54 (24.5)
36-47 months	185	1	0.5	42 (22.7)
48-59 months	160	3	1.9	27 (16.9)
5-9 years	343	2	0.6	39 (11.4)
10+ years	54	0	0	3 (05.5)
Unknown	13	0	0	7 (53.9)
Total	1204	14	1.2	214 (17.7)

CFR = case fatality rate

The present study revealed that only 18% of measles cases had received a measles vaccine. It was noted that the proportion of measles cases that had been been immunized was low in all age groups (15.4% to 22.9%). Out of the 14 fatal cases, only 1 was vaccinated; the rest were not vaccinated against measles. The CFR among vaccinated children was1.4% and in those not vaccinated, it was 0.45%. The CFR was observed to be three times higher among children not vaccinated than those who had been vaccinated [Table 3].

**Table 3:** Case fatality rate in relation to vaccination status

Vaccination status (no.)	Deaths reported	CFR (%)
Vaccinated (220)	1	0.45
Not vaccinated (963)	13	1.35
Unknown (11)	0	0

CFR = case fatality rate

At the time of analysis, the efficacy of the measles vaccination was also calculated. The data for measles immunization coverage was not available for the Shivpuri district. The coverage rate with measles vaccination for the neighboring districts ranged from 29.5% to 47% in various surveys. Shivpuri was presumed to have similar vaccination coverage in the district. Therefore, the vaccine efficacy for 30% coverage is estimated to be 47.7% for children under the age of 5. This was calculated as follows:

VE%= (ARU-ARV)*100/(ARU)

Therefore, vaccine efficacy in this outbreak = ((2828-1478)*100/2828) = 47.7%

Where ARU is the attack rate in unvaccinated children and ARV is the attack rate in those who have previously received 1 dose of measles vaccine.

Out of 1204 cases, complications occurred in 214 (17.7%). The reported complications were highest among an unknown age group (53.9%) followed by the 2-3 years old age group (24.5%;). The most common complication experienced was diarrhea (49.5%), followed by pneumonia (32.7%), and otitis media (13.1%). A few cases (4.67%) also reported other complications, which included encephalitis, laryngotracheobronchitis, etc.

## Discussion

The present study revealed the overall attack rate of measles is 6.2%. It was somewhat higher than the attack rate reported in some other studies in India.(6–8) However, a study carried out in the district of Surat observed an attack rate of 7.6%(10) and another study in Peru reported an attack rate of 29.2% in the same age group.(11) The difference may be attributed to the regional variation and difference in vaccination coverage among the study area and the countries. The higher attack rate was observed in children older than 5 years old (24.2%), which is comparable with the observations made by two other research studies.(7,12) It indicates the accumulation of susceptible population due to low immunization coverage. A higher proportion of males was affected in the present study as compared with females. There are a few studies which have reported similar findings.(13–14) A few other studies have reported no gender difference.(8,15) The reason for the findings may be the difference in the gender ratio in study areas or due to the differential attitude of the parents toward a female child.

The present study indicates the bimodal age distribution of cases with a peak incidence in 2-3 years and in 5-9 years, similar to a study in Harare.(16) This study observed a higher attack rate and CFR for the Shivpuri district in densely populated blocks, as was also reported by Thakur, *et al.*(7) and Kambarami, *et al.*(16) However, the CFR in this study is less than was observed in Uttar Pradesh.(17) This is charecterstic of measles and reflects a need to strengthen routine immunization services. The CFR was higher for females than males, an observation that matches the reported measles outbreak in UP.(17)

The present study revealed that only 18% of the subjects with measles received a measles vaccine. The other study also reported a low vaccination status among measles cases.(8,17) These findings also correspond to the reported immunization coverage in the five poorest states of India.(18) The CFR was also observed to be higher among unvaccinated children compared with vaccinated children. This reflects that measles immunizations not only decrease the attack rate but also the disease severity.(16) The Vitamin A supplementation was also noted to have preventive role in measles related complications in separate section of this study. Those results have already been published elsewhere in an peer reviewed journal.(19)

As the actual immunization coverage was not known for the measles vaccine in the Shivpuri district, coverage of nearby district was taken as a dummy for Shivpuri and at a coverage rate of 30%, vaccine efficacy was calculated to be 47.4%. It was observed that by increasing the vaccine coverage, vaccine efficacy increases. These finding collaborates the finding of a study of Desai,*et al*.(20) In the present study, about 17.7% of the subjects had suffered from a post-measles complication. The most common complication observed in this study was diarrhea (49.5%) followed by pneumonia and Ottis media. The studies have reported complication rates of a similar range with slight differences.(6–8,16,21)

## Conclusions

This study points out that a large proportion of measles cases occur among the unvaccinated population in rural India. The low vaccine coverage put them at risk for repeated outbreaks across the country and the cause for low coverage needs to be studied. Similarly, cases among the higher age group point to an increase in vaccine failure, which may be due to improper cold chain maintenance. The outbreak was reported a long time after the index case in the area. If it had been reported earlier, a number of cases could have been avoided. If India wants to eliminate measles, this study recommends that the issue of improving vaccine coverage maintenance of cold chain needs to be addressed and an effective surveillance system needs to be put into place immediately.

## References

[1] World Health Organisation (2004). Progress in reducing global measles deaths: 1999-2002. Wkly Epidemiol Rec.

[2] Park K (2007). The Textbook of Preventive and Social Medicine.

[3] Centre for Disease Control and prevention (1999). Global Measles control and regional elimination 1998-1999. MMWR Morb Mortal Wkly Rep.

[4] Bhatia V, Swami HM, Rai SR, Gulati S, Verma A, Parasher A (2004). Immunization status in children. Indian Pediatr.

[5] Puri A, Gupta VK, Chakravarti A, Mehra M (2002). Measles vaccine efficacy evaluated by case reference technique. Indian Pediatr.

[6] Sharma MK, Bhatia V, Swami HM (2004). Outbreak of measles amongst vaccinated children in a slum of Chandigarh. Indian J Med Sci.

[7] Thakur JS, Ratho RK, Bhatia SP, Grover R, Issaivanan M, Ahmed B (2002). Measles outbreak in a Periurban area of Chandigarh: Need for improving vaccine coverage and strengthening surveillance. Indian J Pediatr.

[8] Ray SK, Mallik S, Munsi AK, Mitra SP, Baur B, Kumar S (2004). Epidemiological study of measles in slum areas of Kolkata. Indian J Pediatr.

[9] Madhya Pradesh Government Census data of Shivpuri.

[10] (2005). United Nations Children's Fund, National Immunisation Programme, Measles Control: An Urban Challenge.

[11] Sniadack DH, Moscoso B, Anguilar R, Heath J, Bellini W, Chiu MC (1999). Measles epidemiology and outbreak response immunization in a rural community in Peru. Bull World Hlth Organ.

[12] Ratho RK, Misra B, Singh T, Rao P, Kumar R (2005). Measles outbreak in a migrant population. Indian J Pediatr.

[13] Phaneendra Rao RS, Kumari J (1988). Measles in a rural Community. J Commun Dis.

[14] Satpathy SK, Chakraborty AK (1990). Epidemiological study of measles in Singur, West Bengal. J Commun Dis.

[15] Swami SS, Chandra S, Dudani IU, Sharma R, Mathur MM (1987). Epidemiology of measles in Western Rajasthan. J Commun Dis.

[16] Kambarami RA, Nathoo KJ, Nkrumah FK, Pirie DJ (1991). Measles epidemic in Harare, Zimbabwe despite high measles immunization coverage rates. Bull World Hlth Organ.

[17] Singh J, Kumar A (1999). Widespread outbreaks of measles in rural Uttar Prodesh, India 1996: High-risk areas and groups. Indian Pediatr.

[18] Singh P, Yadav RJ (2001). Immunization Status of children in BIMARU States. Indian J Pediatr.

[19] Mishra A, Mishra S, Jain P, Bhadoriya RS, Mishra R, Lahariya C (2008). Measles related complications and the role of Vitamin A supplementation. Indian J Pediatr.

[20] Desai VK, Kapadia SJ, Kumar P, Nirupam S (2002). Impact assessment of mass measles vaccination. Indian J Pediatr.

[21] Bharti B, Bharti S (2002). Measles in hilly hamlet of northern India. Indian J Pediatr.

